# Class II Division 2 subdivision left malocclusion associated with anterior deep overbite in an adult patient with temporomandibular disorder

**DOI:** 10.1590/2177-6709.22.4.102-112.bbo

**Published:** 2017

**Authors:** Ivan Toshio Maruo

**Affiliations:** 1 Professor, Pontifícia Universidade Católica do Paraná, Especialização em Ortodontia (Curitiba/PR, Brasil). Professor, Associação Brasileira de Odontologia do Paraná, Especialização em Ortodontia (Curitiba/PR, Brasil).

**Keywords:** Malocclusion, Angle Class II, Deep overbite, Temporomandibular joint disorders.

## Abstract

The orthodontic treatment of patients with chief complaint of temporomandibular disorders (TMD) presents doubtful prognosis, due to the poor correlation between malocclusions and TMDs. The present case report describes the treatment of an adult patient with Angle Class II Division 2 subdivision left malocclusion associated with anterior deep overbite and TMD. This case was presented to the Brazilian Board of Orthodontics and Dentofacial Orthopedics (BBO), as part of the requirements to obtain the title of BBO Diplomate.

## RESUMO

O tratamento ortodôntico de pacientes com queixa principal de disfunção nas articulações temporomandibulares (DTM) apresenta prognóstico duvidoso, devido à baixa correlação entre as más oclusões e as DTMs. O presente relato de caso descreve o tratamento de um paciente adulto com má oclusão de Classe II, divisão 2, subdivisão esquerda, de Angle e sobremordida profunda, associadas à DTM. Esse caso foi apresentado à Diretoria do Board Brasileiro de Ortodontia e Ortopedia Facial (BBO), como parte dos requisitos para a obtenção do título de Diplomado pelo BBO.

## INTRODUCTION

The patient, a 24-year-old man, attended to the initial appointment, referred by a general dentist. His chief complaints were clicking and occasional pain in temporomandibular joints (TMJs).

He was healthy and presented no significant information in his medical records. In his dental records, biannual attendance to the general dentist, diurnal and nocturnal clenching habits, as well as clicking and occasional pain in his TMJs were noted. Attempts of occlusal adjustment, third molars extractions and the use of interocclusal device to sleep, during approximately two years, were made to lower the symptoms of TMJs pain. However, none of these procedures were successful. 

In functional assessment, mild clicking in both TMJs, during mouth opening and closing movements, indicated a possible “anterior articular disc displacement with reduction”. 

Before any dental intervention, due to the chief complaints, medical assessments by an otorhinolaryngologist, an endocrinologist and a rheumatologist were required, in order to diagnose eventual medical pathologies related to pain in the TMJs region. Nothing was found. 

## DIAGNOSIS

**Figure 1:**
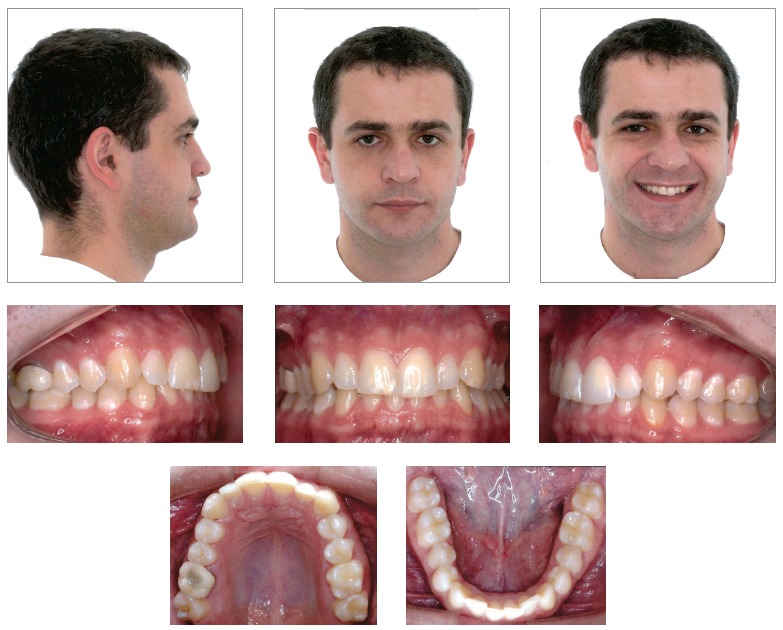
Initial facial and intraoral photographs.

In facial examination (Fig 1), passive lip seal, facial balance and harmony and good profile were noted. The patient presented slight facial asymmetry, with right side more rounded than left side. Nasolabial angle and mentolabial angle were normal. Smile analysis showed adequate maxillary incisors exposure and there was an 1.5-mm maxillary dental midline deviation to the right. As the patient presented nose deviation to the left, there was an impression that the maxillary midline deviation to the right was greater.

**Figure 2:**
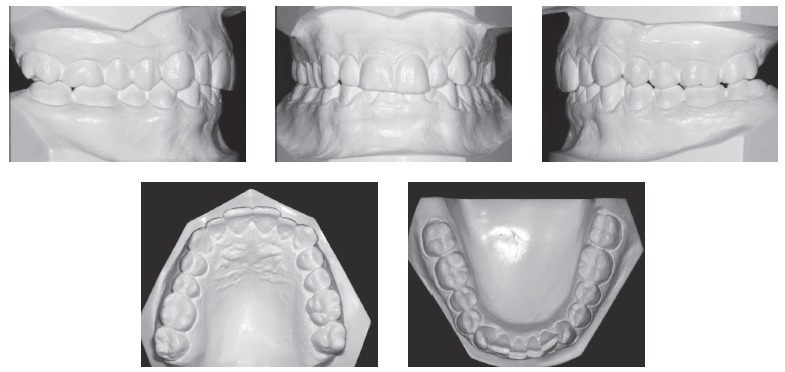
Initial dental casts.

**Figure 3:**
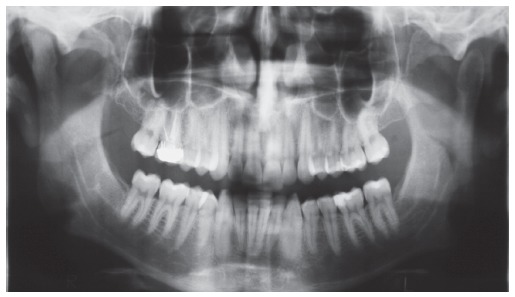
Initial panoramic radiograph.

**Figure 4:**
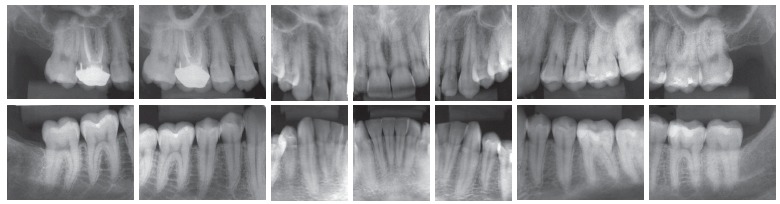
Initial periapical radiographs.

Intraoral examination (Figs 1 and 2) revealed: Class II Division 2 subdivision left, normal overjet, anterior deep overbite, with a deep mandibular curve of Spee, and extruded mandibular incisors and canines, as well as maxillary and mandibular crowding. In relation to the midsagittal plane, dental maxillary midline was 1.5mm deviated to the right and dental mandibular midline was 0.5mm deviated to the left. Besides, maxillary lateral incisors presented size discrepancy and right maxillary first molar was reconstructed with prosthesis.In the panoramic radiograph (Fig 3) and in the periapical radiographs (Fig 4), third molars absence, endodontic treatment and porcelain-fused-to-metal crown on the right maxillary first molar, and restoration on the left mandibular first molar were noted. 

**Figure 5:**
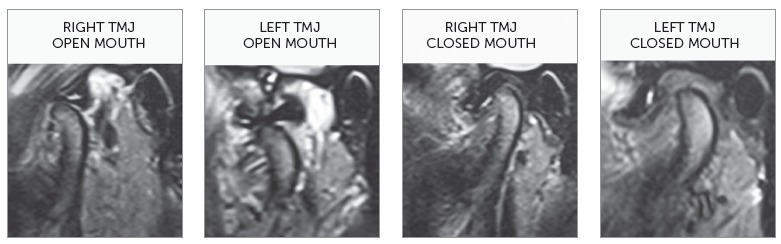
Initial magnetic resonance of right and left TMJs, with opened and closed mouth.

**Figure 6:**
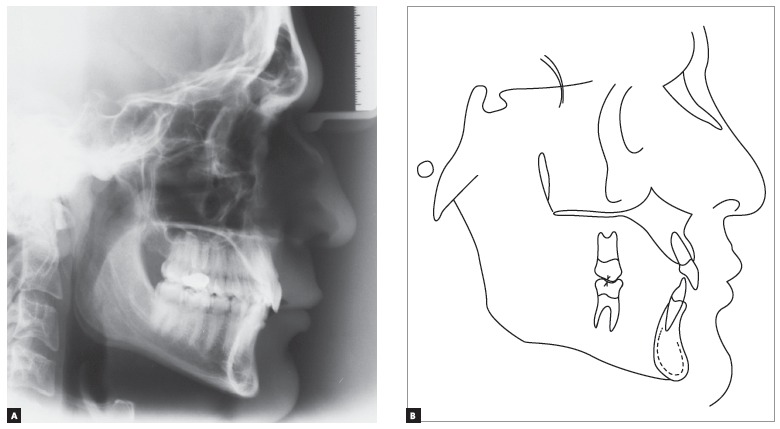
Initial lateral cephalometric radiograph (A) and cephalometric tracing (B).

In order to confirm the clinical diagnosis and to create a baseline for comparison at the end of treatment, magnetic resonance of TMJs was requested. It revealed “anterior articular disc displacement with reduction” in both TMJs. Left displacement was greater than the right one (Fig 5). Cephalometric analysis (Fig 6 and Table 1) showed a brachyfacial skeletal pattern (SN-GoGn = 25^o^; FMA = 17^o^; and Y-Axis = 57^o^) and balanced anteroposterior relationship between the maxilla and the mandible (SNA = 81^o^; SNB = 79.5^o^; and ANB = 1,5^o^), as well as well-positioned maxillary incisors (1.NA = 21^o^, 1-NA = 5.5mm), and retruded and retroclined mandibular incisors (1.NB = 18^o^, and 1-NB = 2.5mm), in relation to their supporting bone bases.

**Table 1:** Baseline (A) and final (B) cephalometric values

	Medidas		Normal	A	B	Dif. A/B
Skeletal pattern	SNA	(Steiner)	82^o^	81^o^	81^o^	0
	SNB	(Steiner)	80^o^	79.5^o^	80^o^	0.5
	ANB	(Steiner)	2^o^	1.5^o^	1^o^	0.5
	Wits	(Jacobson)	♀ 0 ±2 mm ♂ 1 ±2 mm	2 mm	1 mm	1
	Angle of convexity	(Downs)	0^o^	-2^o^	-3^o^	1
	Y-axis	(Downs)	59^o^	57^o^	58^o^	1
	Facial angle	(Downs)	87^o^	91^o^	91^o^	0
	SN-GoGn	(Steiner)	32^o^	25^o^	26^o^	1
	FMA	(Tweed)	25^o^	17^o^	18^o^	1
Dental pattern	IMPA	(Tweed)	90^o^	94^o^	101^o^	7
	1.NA (degrees)	(Steiner)	22^o^	21^o^	25^o^	4
	1-NA (mm)	(Steiner)	4 mm	5.5 mm	6 mm	0.5
	1.NB (degrees)	(Steiner)	25^o^	18^o^	28^o^	10
	1-NB (mm)	(Steiner)	4 mm	2.5 mm	5.5 mm	3
	- Interincisal angle	(Downs)	130^o^	136^o^	122^o^	14
	1-APo	(Ricketts)	1 mm	1 mm	1.5 mm	0.5
Profile	Upper lip - S-line	(Steiner)	0 mm	0 mm	-0.5 mm	0.5
	Lower lip - S-line	(Steiner)	0 mm	-2 mm	-1 mm	1

## TREATMENT PLAN

The treatment objectives were the correction of Class II Division 2 subdivision left, anterior deep overbite, dental midline deviations and crowding.

When explaining to the patient what his orthodontic problems were, it was pointed out that, according to the current literature evidence, there was no guarantee that the correction of his malocclusion would solve his chief complaints of clicking and pain in his TMJs. However, it was also explained that Class II Division 2 subdivision left and anterior deep overbite are contributing factors to increase the pain and discomfort in TMJs.

With these clarifications, the patient decided to undergo orthodontic treatment to improve his dental occlusion, and affirmed that he was fully aware that orthodontic treatment could have no effect in his clicking and occasional pain in the TMJs.

Considering the skeletal bases balance (brachyfacial and Class I skeletal pattern) and the pleasant facial esthetics, the following treatment plans were discussed:

1) Distalization of left maxillary posterior teeth using asymmetric Kloehn low-pull extraoral headgear (HG), until Class II correction, followed by corrective orthodontic treatment, with mandibular incisors and canines intrusion.
2) Aligning and leveling of mandibular and maxillary teeth, followed by the correction of left Class II with temporary anchorage devices (TADs).
3) Extraction of left maxillary first premolar, right maxillary second premolar and mandibular second premolars, followed by corrective orthodontic treatment using Tweed-Merrifield technique, with high-pull “J-hook” extraoral headgear.

Orthodontic treatment involving dental extractions was discarded, because it could flatten the profile and worse the deep overbite, in addition to increasing TMJs pain (TMJs pain was occasional) caused by the “anterior articular disc displacement with reduction” in both TMJs and clenching habits.

After understanding that the orthodontic treatment to correct the left Class II utilizing Kloehn HG would promote more extrusion of posterior teeth than utilizing TADs, and that this extrusion could contribute to decrease the TMD pain, the patient opted for the treatment plan #1, i.e., using asymmetric Kloehn low-pull extraoral HG, followed by corrective orthodontic treatment, with mandibular incisors and canines intrusion.

## TREATMENT PROGRESS

Treatment was initiated with asymmetric HG. Although the left maxillary posterior teeth distalization was taking time, the patient insisted to continue utilizing HG and did not want to use TADs.

When the left Class II was corrected, 0.022 x 0.028-in edgewise standard brackets were bonded on the remaining maxillary teeth, until the correction of the midline deviation. 

Once aligning and leveling of maxillary incisors permitted, 0.022 x 0.028-in edgewise standard brackets were bonded on the mandibular teeth. So, it was possible to align and level mandibular teeth, not only intruding incisors and canines, but also correcting the midline deviation.

As maxillary lateral incisors presented size anomaly, after the brackets and bands removal, the general dentist reshaped these teeth with composite resin.

Fixed mandibular canine-to-canine lingual arch and Hawley maxillary removable appliance were utilized as retention.

## RESULTS 

In the end of orthodontic treatment, the patient reported that he no longer felt pain in the TMJs. So, a new magnetic resonance exam was requested. 

**Figure 7:**
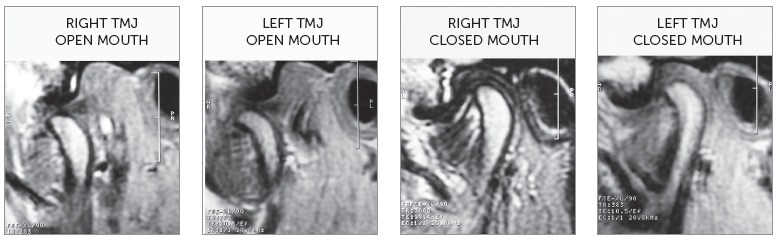
Final magnetic resonance of right and left TMJs, with opened and closed mouth.

The magnetic resonance (Fig. 7) showed that “anterior articular disc displacement with reduction” in both TMJs persisted. This finding was explained to the patient, who understood the need of monitoring his clenching habit.

**Figure 8:**
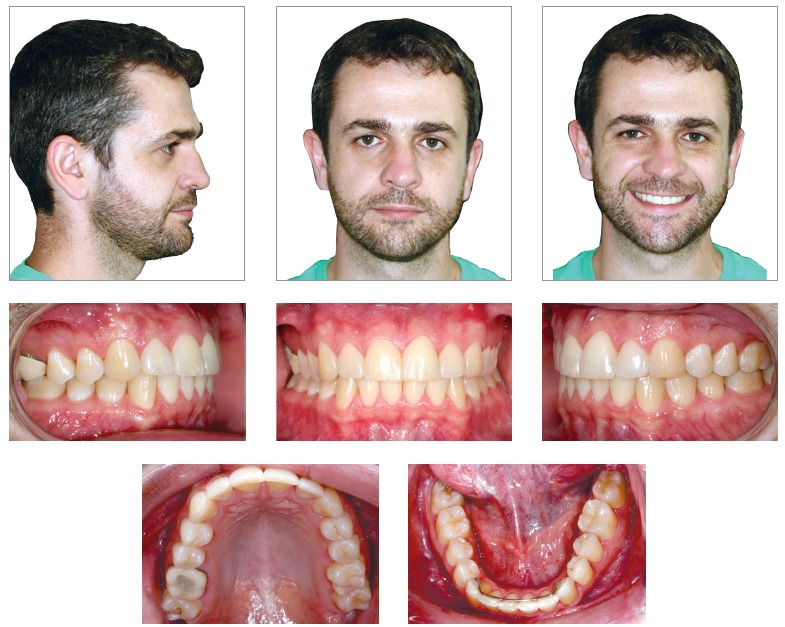
Final facial and intraoral photographs.

Final records showed maintenance of facial balance (Fig 8), with passive lip seal, normal nasolabial angle and mentolabial angle, as well as good profile. There was no change in the slight asymmetry (right side more rounded than left side). When smiling, adequate maxillary incisors exposure was preserved, and the maxillary midline right deviation was corrected. As the discreet nose deviation to the left persisted, it caused the impression that slight maxillary and mandibular midline deviations in relation to the midsagittal plane were present. 

**Figure 9:**
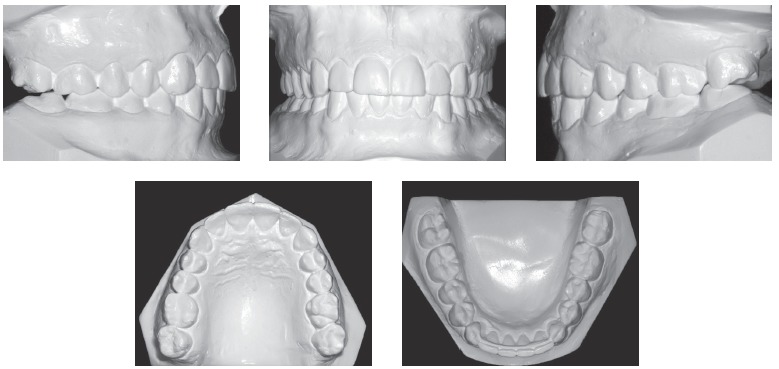
Final dental casts.

With the performed orthodontic treatment, the anteroposterior relationship between left posterior teeth, the overbite as well as the maxillary and mandibular midline deviations were corrected, and the normal overjet was maintained (Figs 8 and 9).

**Figure 10:**
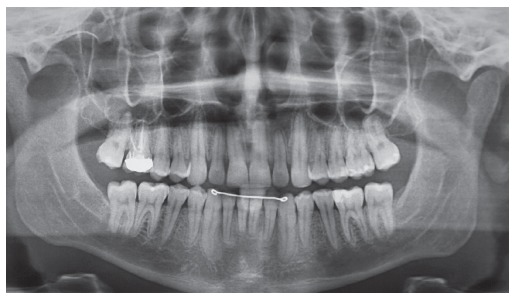
Final panoramic radiograph.

**Figure 11:**
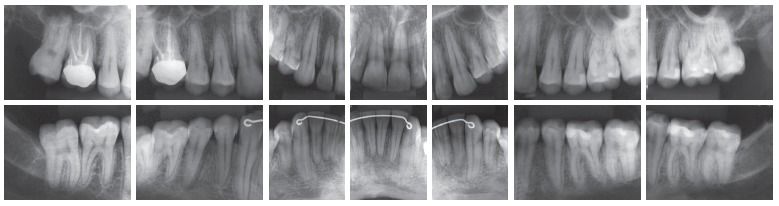
Final periapical radiographs.

In the panoramic radiograph (Fig 10) and in the periapical radiographs (Fig 11), no alterations were verified. Dental and periodontal health were maintained. 

**Figure 12:**
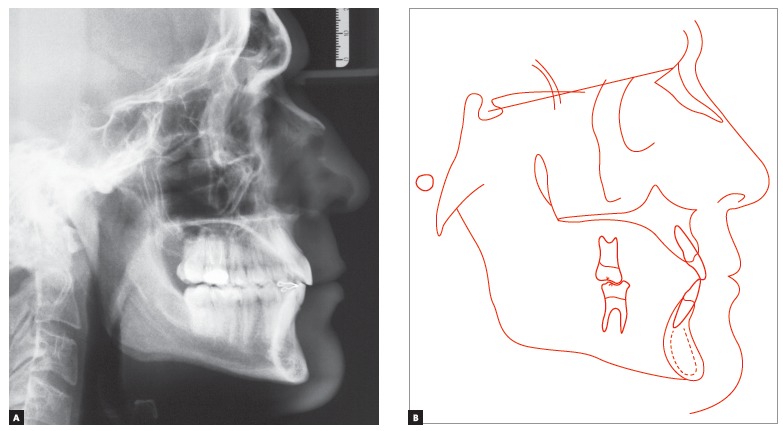
Final lateral cephalometric radiograph (A) and cephalometric tracing (B).

Final cephalometric analysis (Fig 12, Table 1) showed a slight mandibular plane opening (SN-GoGn = 26^o^; FMA = 18^o^; and Y-axis = 58^o^), and maintenance of a balanced anteroposterior relationship between maxilla and mandible (SNA = 81^o^; SNB = 80^o^; and ANB = 1^o^). Besides, mandibular incisors were buccally inclined (1.NB = 28^o^ and 1-NB = 5.5mm), and maxillary incisors presented with a discreet buccal inclination (1.NA = 25^o^ and 1-NA = 6mm). 

**Figure 13:**
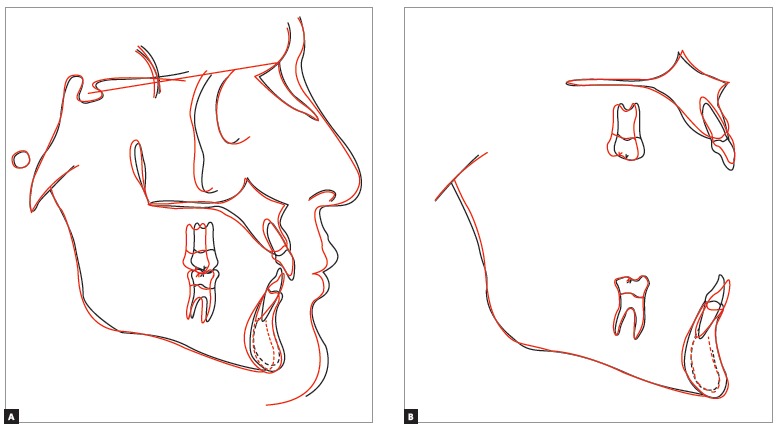
Total superimposition (A), maxillary and mandibular superimpositions (B) of initial (black) and final (red) cephalometric tracing.

Total cephalometric superimposition (Fig 13) demonstrated a slight mandibular plane opening, due to the left maxillary posterior teeth distalization, and a discreet improvement of facial lower profile, caused by the improved balance of upper and lower lips.

Maxillary cephalometric superimposition (Fig 13) showed left maxillary molar distalization, without extrusion, and intrusion of maxillary incisors, with lingual root torque and with crowns maintaining their anteroposterior position.

Mandibular cephalometric superimposition (Fig 13) evinced that anteroposterior and vertical position of left mandibular molar was maintained. Besides, mandibular incisors were intruded and buccaly inclined, with their crowns moving buccally and their roots moving lingually. 

## FINAL CONSIDERATIONS

Orthodontic treatment of patients with chief complaint of temporomandibular disorders (TMD) is complex, because this dysfunction has many etiological factors and the literature in this subject is controversial. According to some authors, it is not possible to affirm that malocclusions are an etiological factor of TMD^1^ ^-^ ^3^, while other authors claim the opposite.^6^ Additionally, it is not possible to affirm that orthodontics plays a relevant etiological or therapeutic role in TMDs.^4^ ^,^ ^5^

Because of that, it is important that, before any dental intervention, differential diagnosis is made, requiring medical assessment to rule out the possibility of presence of systemic pathologies with similar TMD symptoms, as gout,^7^ osteosarcoma^8^ and pseudotumor^9^ in TMJs, Eagle’s syndrome,^10^ fibromyalgia,^11^ rheumatoid arthritis^12^ and trigeminal neuralgia.^13^

Following this principle, in this case, any other medical pathology which could cause similar TMDs symptoms was excluded. 

Considering both the clicking in the end of mouth opening and closing movements, and the magnetic resonance images, the diagnosis of “anterior articular disc displacement with reduction” was reached. This is an intracapsular disorder, in which the disc is in an anterior position relative to the condylar head, in the closed mouth position, and the disc reduces upon opening of the mouth.^14^

Faced with this condition, it was verified that the cause of the occasional pain symptoms could be related to the “anterior articular disc displacement with reduction” associated with the clenching habit. The presence of a Class II relationship of left posterior teeth and an anterior deep bite could contribute to increase pain and discomfort of TMJs. As Costa et al^15^ verified, there is correlation among TMD, deep overbite and Class II malocclusion, when these variables are associated with clenching habit, although is not possible to affirm if occlusal factors are predisposing, precipitating or perpetuating the disease. 

Considering the malocclusion, the orthodontic treatment could be with or without extractions, utilizing either HG or TADs. However, as there was the possibility of overbite increase, that would accentuate TMD symptoms, and because of the pleasant profile, the chosen treatment plan was without extractions.

The choice for HG utilization, instead of TADs, in the treatment, aimed to act not only in the malocclusion but also in the TMD.

There is not enough evidence to measure the effects of orthodontic treatment in the signs and symptoms of TMD.^16^ However, there are studies that point out that orthodontic appliances are as effective as interoclusal devices, in the treatment of pain in “anterior articular disc displacement with reduction” cases.^17^

In this case, the utilization of Kloehn HG aimed to take advantage of its potential to distalize and extrude molars.^18^ If both of these movements occurred, not only the left Class II malocclusion would be corrected, but also the TMD symptoms would be relieved, through the seven common features of conventional interocclusal devices, described by Okeson^19^: 1) change of occlusal conditions; 2) change of condylar position; 3) increase in vertical dimension; 4) cognitive sensitization; 5) placebo effect; 6) increase in peripheral stimuli to central nervous system; and 7) symptoms regression to the mean. 

The goals of TMD treatment and malocclusion correction were achieved. There was remission of chief complaints of clicking and discomfort in the TMJs. Left Class II, deep overbite, right maxillary midline deviation and left mandibular midline deviation were corrected and the normal overjet was maintained. Functional harmony during protrusive, as well as right and left lateral movements, was obtained. Pleasant facial profile was maintained and there was only slight change in vertical dimension. In this case, there was no concern about third molars, because they had been extracted in an attempt to solve the functional problems. In the end of orthodontic treatment, as the patient reported that he no longer felt pain in the TMJs, a new magnetic resonance was requested. There was the expectation that the TMJs articular discs position could have been corrected. Unfortunately, the magnetic resonance image showed the maintenance of the “anterior articular disc displacement with reduction” in both TMJs, practically with the same severity identified in the initial magnetic resonance.

Possibly, although in a short period of time, the remission of pain symptoms could have been influenced by the process of retrodiscal tissue fibrosis, which created a “pseudodisc” over the condyle head,^20^ and provided protection to previously damaged structures.

Therefore, the corrective orthodontic treatment can be considered successful, because the facial balance of the patient was maintained, the initial dimensions of dental arches were respected, good dental intercuspation was achieved, and mandibular movements with immediate desocclusion were established. 
